# The Microbial Hypothesis: Contributions of Adenovirus Infection and Metabolic Endotoxaemia to the Pathogenesis of Obesity

**DOI:** 10.1155/2016/7030795

**Published:** 2016-11-24

**Authors:** Amos Tambo, Mohsin H. K. Roshan, Nikolai P. Pace

**Affiliations:** Centre for Molecular Medicine and Biobanking, University of Malta, Msida, Malta

## Abstract

The global obesity epidemic, dubbed “globesity” by the World Health Organisation, is a pressing public health issue. The aetiology of obesity is multifactorial incorporating both genetic and environmental factors. Recently, epidemiological studies have observed an association between microbes and obesity. Obesity-promoting microbiome and resultant gut barrier disintegration have been implicated as key factors facilitating metabolic endotoxaemia. This is an influx of bacterial endotoxins into the systemic circulation, believed to underpin obesity pathogenesis. Adipocyte dysfunction and subsequent adipokine secretion characterised by low grade inflammation, were conventionally attributed to persistent hyperlipidaemia. They were thought of as pivotal in perpetuating obesity. It is now debated whether infection and endotoxaemia are also implicated in initiating and perpetuating low grade inflammation. The fact that obesity has a prevalence of over 600 million and serves as a risk factor for chronic diseases including cardiovascular disease and type 2 diabetes mellitus is testament to the importance of exploring the role of microbes in obesity pathobiology. It is on this basis that Massachusetts General Hospital is sponsoring the Faecal Microbiota Transplant for Obesity and Metabolism clinical trial, to study the impact of microbiome composition on weight. The association of microbes with obesity, namely, adenovirus infection and metabolic endotoxaemia, is reviewed.

## 1. Introduction

Obesity is a chronic multifactorial pathology driven by the complex interaction between various factors. Extensive research has implicated a number of genetic, epigenetic, nutritional, and lifestyle factors. Furthermore, the composition of the gut microbiome has also been implicated in the development of obesity. Irrespective of the specific causes leading to obesity, it is a strong risk factor for the development of cardiometabolic diseases, including insulin resistance, type 2 diabetes, and hypertension. The clinical consequences of obesity extend further to include a spectrum of other diseases such as malignancies, osteoarthritis, and obstructive sleep apnoea [1, 2]. Moreover, obesity exerts a heavy toll in terms of social and economic consequences [3, 4]. The rising prevalence of both childhood and adult obesity is strongly linked to caloric excess and the sedentary urban lifestyle typical of “Westernised” countries [5]. There is, however, growing evidence for a robust physiological process that restricts the voluntary mechanisms to reduce body weight by drastic changes in lifestyle. In this respect, obesity is increasingly recognised as a disease rather than as a wilful choice [6, 7].

Viral infection has been implicated as a contributing factor in the aetiology of obesity in both animal and human studies [8]. In addition, the gastrointestinal tract is home to over 10^14^ bacteria that collectively form the intestinal microbiome, with a joint genetic repertoire that is larger than the human genome [9]. These symbiotic bacteria establish and maintain the gut immune system and contribute to the breakdown of complex nondigestible plant-derived polysaccharides [10, 11]. Recent evidence links the gut microbiome to the development of obesity and metabolic disease [12]. The aim of this review is to present the epidemiological evidence for the association between microbes and obesity, and discuss the cellular and molecular mechanisms underpinning the inflammatory response that is central to adipocyte dysfunction in obesity pathobiology.

## 2. Conventional Perspective of Adipocyte Dysfunction in Obesity Pathogenesis

### 2.1. Adipocyte Stress Response

Increased circulating levels of triglycerides and glucose perturb metabolic homeostasis and stimulate compensatory adipocyte hypertrophy and hyperplasia [13]. Persistently elevated triglyceride and glucose levels overwhelm the physiological response such that decompensation (so-called adipocyte stress response) arises, and thus adipocyte dysfunction ensues [14–16].

### 2.2. Chronic Low Grade Inflammation in Adipocyte Dysfunction

Adipocyte dysfunction is epitomised by a low grade chronic inflammatory response, which is an integral feature of obesity [20, 21]. It has been hypothesised that increased caloric intake triggers inflammation leading to adipocyte dysfunction [16]. Adipose tissue is infiltrated with bone marrow-derived macrophages, and the density of these adipose tissue macrophages (ATMs) is dependent on the degree of obesity. The number of ATMs correlates with the degree of insulin resistance [22]. Adipose tissue macrophages exhibit different phenotypes depending on the degree of adiposity. ATMs in lean nonobese individuals are anti-inflammatory (M2 macrophages) as opposed to ATMs in obese individuals that express proinflammatory genes (M1 “classically-activated” macrophages) [23].

In nonobese, insulin sensitive conditions, adipocytes secrete factors that trigger alternative activation of macrophages. The obesity-induced changes in adipocyte gene expression trigger release of proinflammatory cytokines (TNF-*α*, MCP1) that lead to recruitment and activation of macrophages. The activated M1 macrophages produce and secrete other proinflammatory mediators, including IL-6, TNF-*α*, IL-1*β*, and resistin. These mediators establish a positive feedback loop that further enhances insulin resistance and inflammation [23]. Cytokines such as TNF*α*, IL-6, and IL-1*β* exert paracrine effects to activate inflammatory pathways. This leads to activation of Jun N-terminal kinase (JNK), inhibitor of *κ* B kinase *β* (IKK), and other serine kinases, which activate transcription factor targets (c-Fos/c-Jun) and nuclear factor-kB (NF-kB) that stimulate transcription of inflammatory pathway genes [24, 25].

IL-6 secreted by adipose tissue macrophages is considered a major inflammatory mediator. Primarily, IL-6 triggers subclinical inflammation by instituting a vicious cycle that includes the downregulation of lipoprotein transport to peripheral tissues. This in turn increases plasma lipid levels and promotes triglyceride release into the circulation. The result is development of overt hypertriglyceridemia and hyperlipidaemia, which serves to perpetuate the obesity phenotype by presenting a constitutive signal for adipocyte hypertrophy and hyperplasia. Adipocyte dysfunction thus ensues. It has also been shown that IL-6 expression is more pronounced in metabolically active visceral rather than peripheral adipocytes [26].

### 2.3. Adipokine Secretion

The adipocyte plays a key endocrine role in the secretion of adipokines. This topic has been extensively reviewed by Ouchi et al. [27]. Adipokine secretion is dysregulated in obesity with increased levels of obesity-promoting adipokines. The expression of proinflammatory proteins, particularly TNF-*α*, may lead to a reduction in anti-inflammatory adipokines, such as adiponectin which plays a pivotal anti-inflammatory role [28]. Despite the extensive research on adipose tissue inflammation, the direct causal link between the events that trigger chronic inflammation and the final metabolic outcome is still not clearly determined. This review will focus on presenting the evidence linking microbes to adipose tissue dysfunction in the setting of obesity.

## 3. Viral Infection and Obesity

### 3.1. Animal Viruses and Obesity

The first association between viruses and obesity in animals was reported by Lyons et al. Mice infected with canine distemper virus (CDV) demonstrated adipocyte hypertrophy and hyperplasia in the absence of CNS pathology [29]. CDV does not infect humans but is antigenically related to the* Paramyxoviridae* family that includes the measles virus. Subsequently other investigators had demonstrated that the virus targets specific hypothalamic regions and is associated with alteration of various neuropeptide-signalling pathways, including leptin, neuropeptide Y, and melanin-concentrating hormone [30–33]. Rous-associated virus 7 (RAV-7) has also been associated with a syndrome of obesity, stunted growth, hypercholesterolaemia, fatty liver, and thyroid dysfunction in chickens [34]. Borna disease virus (BDV) has similarly been implicated in rat obesity. This RNA virus causes a lymphomonocytic inflammation of the hypothalamus, pancreatic islet cell hyperplasia, and elevations of serum glucose and triglyceride levels [35, 36]. BDV is neurotropic and has a wide range of animal hosts. Seroepidemiological studies have implicated BDV in neuropsychiatric diseases including schizophrenia and depression in some populations, though the associations have not been consistent [37–39].

Another animal model of virus-induced obesity implicated the SMAM-1 avian adenovirus in chickens. SMAM-1 was shown to lead to increased adiposity independent of food intake [40]. Subsequently, Dhurandhar et al. demonstrated that body weight and BMI were higher in human subjects positive for anti-SMAM-1 antibodies compared to those negative for anti-SMAM-1 antibodies [41]. This was the first reported association of viral infection with human obesity. The transmissible spongiform encephalopathy scrapie that infects cattle has also been linked to obesity. Kim et al. showed that inoculation of mice with the ME7 strain of scrapie results in obesity that is dependent on the hypothalamic-pituitary-adrenal axis [42].

The few associations between viral infection and animal obesity described above are clearly limited in translational terms, yet are still crucial in demonstrating a strong association between viral infection and obesity. Human adenovirus 36 (Ad-36) is a common human pathogen that has been linked to human obesity and adipocyte function through different mechanisms and is discussed next.

### 3.2. Adenovirus 36 and Obesity—*In Vivo* Studies

The adenoviruses are an important group of widely prevalent human pathogens that have been extensively studied. They are non-enveloped icosahedral DNA viruses with a linear double stranded genome that can infect a wide range of species and tissues. Fifty-two human adenovirus serotypes have been described. Their structural biology, genome, evolution, and host cell immune response has been reviewed elsewhere [43–45]. Clinically, they are responsible for a wide array of self-limiting infections, primarily of the respiratory and gastrointestinal tracts. Furthermore, they are effective vectors for gene-based immunotherapy and gene replacement strategies [46]. Ad-36 has been associated with obesity in both animal and human studies.

Ad-36 was first isolated from the faeces of a girl with enteritis [47]. Genomic characterisation of the virus revealed several unique features compared to other human adenoviruses that could possibly account for its unique tissue tropism [48]. The first reported link between Ad-36 and obesity was described in the year 2000 in animal models, where chicken and mice inoculated with Ad-36 demonstrated increased adipose tissue and decreased cholesterol and triglyceride levels in the absence of CNS histopathological changes [49]. Ad-36 DNA was detected in adipose tissue but not in skeletal muscle of the inoculated animals. Subsequently, other studies reproduced the adiposity-promoting effects of Ad-36 in two non-human primate models [50]. It was also shown that horizontal transmission of Ad-36 from infected to control animals produces adiposity and is accompanied by a paradoxical reduction in serum lipids [51]. Rats infected with Ad-36 demonstrate an increase in weight and insulin sensitivity, visceral adipose tissue expression of glycerol 3-phosphate dehydrogenase, and peroxisome proliferator-activated receptor gamma (PPAR-*γ*). CCAAT/enhancer-binding protein (C/EBP) alpha and beta were greater in infected animals compared to controls [52]. Kapila et al. reported that in a hamster model Ad-36 infection altered lipoprotein metabolism to produce a more atherogenic profile, with diminished HDL-C and increased LDL-C [53]. Viral DNA as a result of acute Ad-36 infection is detected in multiple tissues early following inoculation [54].

### 3.3. Adenovirus and Adipocyte Biology

Vangipuram et al. provided the first insight into the molecular mechanisms underpinning Ad-36-induced adipogenesis. The authors demonstrated that viral infection in 3T3-L1 murine preadipocytes induces differentiation and an increase in total cell lipid content [55]. These features are not shared with human adenovirus 2. Subsequently, Rathod et al. showed that transient Ad-36 mRNA expression, but not viral DNA replication, is required for preadipocyte differentiation [56]. The increased expression of PPAR-*γ* and C/EBP reported in animal studies is related to the proadipogenic effects observed* in vitro*, as these genes are key transcription factors that regulate adipocyte differentiation [52, 57]. A detailed analysis of the cellular pathways involved in Ad-36-induced adipogenesis showed that the viral E4 orf-1 gene stimulates proadipogenic differentiation in both 3T3-L1 and human adipose stem cells [58]. Furthermore, Ad-36 E4 orf-1 modulates insulin sensitivity via activation of the phosphatidylinositol 3-kinase (PI3 K) pathway. This accounts for the observed reduction in fasting insulin levels [52]. Studies also showed that Ad-36 suppresses the expression of leptin mRNA and improves glucose uptake in adipocytes [59]. The role of Ad-36 in human adipose tissue was further reinforced by studies that demonstrated that the virus induces commitment, differentiation, and lipid accumulation in human adipose-derived stem cells [60]. Adipocyte differentiation and lipid accumulation were observed in the absence of inducers of adipogenesis and in osteogenic culture media. They were accompanied by expression of the adipocyte-specific transcription factors PPAR-*γ* and C/EBP*β*. Wang et al. also demonstrated that Ad-36 significantly reduced fatty acid oxidation and increased de novo lipogenesis in cultured human muscle cells [61]. Bouwman et al. further demonstrated* in vitro *that incubating preadipocytes and adipocytes with adenovirus (subtypes 2 and 36), amongst other viruses, was responsible for increased production of IL-6 which might contribute to chronic low grade inflammation [62].

## 4. Adenovirus and Obesity—Epidemiological Links

A number of investigators have studied the epidemiologic association between Ad-36 infection and human obesity. These investigations were carried out in different age groups and population ethnicities and utilised various methodologies for viral detection. The salient findings of these human seroepidemiologic studies have been recently described by Ponterio and Gnessi [63]. The unique genomic and proteomic structure of Ad-36 limits antibody cross reactivity in serum neutralization assays and further strengthens the reported associations between Ad-36 and human obesity. The majority, though not all studies, showed greater seropositivity for Ad-36 in obesity, with extensive variation in seroprevalence rates between countries. Three meta-analyses also showed an association between Ad-36 infection and obesity in both adults and children and are summarized in Table 1.

## 5. Gut Microbiome, Gut Barrier Integrity, and Their Role in Obesity Pathogenesis

The role of bacteria and their associated products in the pathogenesis of obesity has recently attracted considerable interest. Moreover, it has been proposed that the composition of the gut microbiome may contribute to obesity through their effects on the gut barrier and beyond.

### 5.1. Gut Microbiome

The relatively recent technological advances in genomics have revolutionized the study of the intestinal microbiome. It is now possible to sequence mixed microbial genetic material directly extracted from environmental samples without prior laboratory culture of individual species. This emerging field, known as metagenomics, enables a survey of the different microorganisms present in a specific environment [64]. Several large-scale projects such as the Human Microbiome Project have characterised microbial genomes from hundreds of isolated human symbionts and have shed light on the complex interplay between the human host and its microbial populace. The implications of compositional changes in health and disease have also been elucidated (http://hmpdacc.org/). Butyrate along with propionate and acetate are short-chain fatty acids (SCFAs) derived from the bacterial degradation of complex polysaccharides in the gut [65]. They have important metabolic roles with butyrate acting as a metabolic substrate for colonic epithelial cells. The functions mediated by butyrate that are pertinent to the pathogenesis of obesity include its role in the maintenance of intestinal epithelial integrity, thereby preventing the translocation of endotoxins produced by intestinal Gram-negative bacteria across the gut barrier [66].

### 5.2. The Gut Barrier

Maintaining the integrity of the gut barrier is critical in preventing contact between materials originating from the nonsterile external environment (gut lumen) with the sterile systemic circulation [67]. Immunoprotection is conferred by means of the gut-associated lymphoid tissue (GALT) that is present on the mucosal surface and contains antigen presenting cells capable of processing and presenting exogenous antigens. The purpose of antigen presentation is primarily to activate adaptive immunity [68]. The gut barrier is composed primarily of enterocytes which adhere to one another by means of tight junctions, desmosomes, and adherens junctions [69]. This is important in order for discriminative transport of material to occur effectively between the gut lumen and the systemic circulation. Moreover, the gut barrier's role as a physical barrier to infection is key in preventing dissemination of bacteria and their associated products from the lumen into the systemic circulation [69]. The integral membrane proteins claudins and occludin, along with cytoskeletal adaptor proteins ZO-1, ZO-2, and ZO-3, have been demonstrated to facilitate paracellular transport between enterocytes [70]. Disruption of the gut barrier at any of these points results in indiscriminate dysregulated paracellular transport [67, 71]. Furthermore, it has been suggested that the integrity of the tight junction barrier is under strict regulation of proinflammatory and anti-inflammatory signalling. Proinflammatory cytokines implicated in promoting tight junction leakage include TNF-*α* and IFN-*γ*. Contrarily, anti-inflammatory cytokines including TGF-*β* and IL-10 have been suggested to maintain the integrity of tight junctions by downregulating proinflammatory signalling [72–76].

### 5.3. Dysbiosis

Failure to maintain a robust gut barrier, termed gut barrier dysfunction, may lead to an altered microbiome composition, resulting in microbiome imbalance. This is known as dysbiosis [67, 77]. The underlying causes of dysbiosis are diverse, and a high fat diet poor in prebiotic-containing nutrients has also been highlighted as a leading cause [78]. A high fat diet has been shown to increase the ratio of obesity-promoting bacteria in the microbiome [79].

*In vivo* studies have been conducted to demonstrate the importance of gut microbiome composition. Widely used models include the obesity mouse model* ob/ob (*leptin deficient) and the* db/db* (leptin receptor mutant) mouse. In a study conducted by Turnbaugh et al. it was shown that transfecting sterile laboratory mice with microbiota from* ob/ob *resulted in the expression of the obesity phenotype akin to* ob/ob* mice [80]. Moreover, sequencing studies performed by Ley et al. compared the microbiome of* ob/ob* mice and lean wild type mice. This revealed changes in two dominant bacterial divisions: Bacteriodetes and Firmicutes. Firmicutes levels were found to be increased in comparison to Bacteriodetes in* ob/ob *mice [81].

The situation is parallel in humans. Ley et al. extended their study to human subjects who adopted either a low-carbohydrate or a low-fat diet for weight control purposes, during a one-year period. Follow-up during this period demonstrated that a change in Bacteriodetes and Firmicutes levels had occurred in study subjects compared to controls. The change was such that from an initial elevated baseline Firmicutes : Bacteriodetes ratio, the ratio had normalised in those subjects who lost weight and maintained a lean body weight after a year, matching that of controls [82]. Furthermore, faecal microbiome studies in lean and obese monozygotic and dizygotic twins established that the microbiome is shared between relatives, though bacterial lineages were individual-specific. A single dominant bacterial species was not identified and it was concluded that the core microbiome is shared in terms of genetic similarity rather than at a dominant organismal level [83]. Certain studies in humans have revealed beneficial commensal bacteria that strive to maintain the integrity of enterocytes and thus the gut barrier. These include* Akkermansia muciniphila* found in the mucosal mucus coating as well as* Faecalibacterium prausnitzii* [84, 85]. In dysbiosis such beneficial organisms are lost, and this is coupled with endotoxin secretion by other harmful bacteria that promote gut barrier breakdown, precipitating metabolic endotoxaemia.

### 5.4. Metabolic Endotoxaemia

Central to the paradigm of bacteria as contributors to the pathogenesis of obesity is the concept of “metabolic endotoxaemia.” Put simply, this is increased plasma bacterial lipopolysaccharide (LPS) levels which are believed to originate from Gram-negative gut microbiome that spill into the systemic circulation as a direct result of gut barrier dysfunction [67, 77, 78]. A number of systems have been implicated in mediating the “gut-to-adipocyte axis” signalling, a term that we have coined to describe the communication between the gut microbiome and their associated endotoxins with adipocytes. Some systems have been proposed to function as gatekeepers and others as mediators of toxin spillage in the setting of gut barrier dysfunction. These systems include the endocannabinoid system, the apelinergic system, and the enteroendocrine system [78, 86].

#### 5.4.1. Endocannabinoid System

The endocannabinoid system is a key mediator of molecular signalling between LPS (chiefly originating from Gram-negative organisms) and adipocytes [78, 87]. The two most studied bioactive lipids are N-arachidonoylethanolamine (AEA) and 2-arachidonoylglycerol (2-AG) which activate the G protein-coupled receptors CB1 and CB2 [88]. Though the expression of CB1 and CB2 was traditionally believed to be limited to the central nervous system, recent evidence suggests that gastrointestinal expression also occurs [89].* In vivo* and* ex vivo* studies have implicated the CB1 receptor in gut-to-adipocyte signalling. The exact molecular signalling pathway that mediates metabolic endotoxaemia has not yet been elucidated [88, 90]. However, CB1 receptor activation has been shown to modulate ion channels and activate different mitogen-activated protein kinase (MAPK) pathways (p42/p44 MAPK, p38 MAPK, and c-Jun N-terminal kinase). It has also been implicated in the inhibition of adenylyl cyclase in tissues and cells. The aforementioned actions have been attributed to G_i/o_ proteins. In the case of MAPK pathways, aside from G_i/o_ proteins, PI3 K also mediates CB1 receptor activation [91]. Furthermore, CB1 receptor stimulation results in activation of inwardly rectifying K^+^ channels [92]. Similarly, L-type voltage-gated calcium channel activity was also shown to be targeted by CB1 receptor activation in cat cerebral arterial muscle and neonatal rat nucleus tractus solitarius. The effects here were primarily inhibitory [93, 94].

Among the wide array of physiological effects mediated by the endocannabinoid system, the one most pertinent to obesity is mainly increased energy intake and storage [95, 96]. Interestingly, the administration of the CB1 antagonist SR141716 in humans is accompanied by decreased body weight and anorexigenic effects [78]. Furthermore, to investigate the manner in which CB1 receptor agonist treatment or LPS treatment gives rise to the metabolic features of diet-induced obesity, mice were chronically treated with low-dose LPS before and during a high fat challenge. The plasma LPS levels were significantly increased (metabolic endotoxaemia) as well as mRNA levels of the macrophage markers* F4/80* and* CD11c* in adipose tissue. The increased macrophage mRNA markers suggested macrophage infiltration in adipose tissue [90]. This study clearly demonstrates a cause and effect relationship between LPS inoculation and mediators of inflammation.

It is therefore believed that one of the manners by which endotoxaemia promotes obesity is through the upregulation of the endocannabinoid system, thereby indirectly promoting adipogenesis [97]. Endotoxaemia has also been implicated in setting up chronic low grade inflammation in obesity, which is in keeping with experimental evidence in animal models [78].

#### 5.4.2. Apelinergic System

Apelin is a peptide involved in the regulation of the central nervous, gastrointestinal, cardiovascular, and immune systems. It is also an adipokine secreted by mature adipocytes in mice and humans and mediates its effects via the APJ receptor [98]. It has been suggested as a negative regulator of obesity, though its precise mechanisms in this capacity are yet to be unravelled [87]. A myriad of signalling cascades are triggered upon activation of APJ receptor, the endpoint of which is the activation or repression of a number of transcription factors. Coupling of the APJ receptor to G_q/11_ stimulates PLC-*β* signalling and subsequent hydrolysis of phosphatidylinositol 4,5-bisphosphate (PIP_2_) to IP_3_ and diacylglycerol (DAG). DAG activates PKC which then activates the Ras protein. The Ras cascade can proceed by activating either the MAPK or JNK pathways. An alternative signalling pathway also exists, where the APJ receptor is coupled to G_i/o_. This pathway activates the MAPK cascade via PKC and it can also activate PI3K, which in turn activates Akt and mammalian target of rapamycin (mTOR). mTOR activates both p70S6K and endothelial nitric oxide synthase (eNOS). In addition, adenylate cyclase is inhibited by G_i/o_, whereas G_s_ increases cyclic AMP synthesis from ATP and activates the PKA pathway [99].

A hyperactive endocannabinoid system in the pathogenic setting of obesity, along with low grade chronic inflammation, has been suggested as the culprits for the augmentation in apelin levels observed in obese animal models and humans [87]. The mechanisms of communication between the endocannabinoid and apelinergic systems are poorly understood. It has been previously shown that inflammation regulates both apelin and APJ mRNA expression [87]. Intraperitoneal administration of apelin in obese and normal mice over a 14-day period induced weight loss independent of food intake [100]. In addition, it was demonstrated in* db/db *mice that apelin and APJ mRNA levels showed twofold and threefold respective increase [87]. Possibly, a hyperactive endocannabinoid system and low grade inflammation may directly or indirectly trigger activation of the apelinergic system as a homeostatic mechanism to counteract obesity.

#### 5.4.3. Enteroendocrine System

The L-cells of the enteroendocrine system are neuroendocrine cells expressed from the duodenum to the rectum and produce glucagon-like peptides 1 and 2 (GLP-1 and GLP-2) [101, 102]. GLP-1 is an incretin hormone known for its role in glucose homeostasis and energy balance. It also inhibits glucagon secretion, reduces food intake, and delays gastric emptying. Its role extends beyond glucose homeostasis [102]. GLP-1 may be implicated in antagonising the effects of metabolic endotoxaemia by regulating epithelial cell proliferation, thus sustaining the gut barrier [103]. In order to determine the mechanisms through which intestinal proliferation is promoted by GLP-1, C57BL/6 WT mice were treated with the GLP-1 receptor agonist exenatide (EX-4: exendin-4) for a period of one week. Increased weight, length, and weight per unit length of small bowel and large were noted. These effects were also observed with chronic (1 month) treatment of C57BL/6 WT mice. However, this effect was not evident in *Glp1*r^−/−^ mice who were subjected to the same treatment over the same period of time. Upregulated gene expression profiles included* IGF1*,* IGF1R*, and* areg*. A conditional deletion of the* IGF1R* gene from intestinal epithelial cells and subsequent Liraglutide and Exenatide administration increased both small bowel and colon weight and length. Activation of GLP-1 receptor signalling in Fgf7^−/−^ mice failed to increase small bowel and colon mass in the absence of Fgf7. It was therefore deduced that GLP-1 receptor agonists activate components of IGF-1 and ErbB signalling pathways and that the proliferative actions of GLP-1 receptor agonists are selectively mediated through mechanisms requiring Fgf7 [102].

Similarly, GLP-2 has been shown to induce proliferation* in vitro* in Caco-2 cells. The cells were subcultured in the presence or absence of GLP-2 as well as different kinase antagonist concentrations. These included Genistein (global tyrosine kinase inhibitor), LY294002 (PI3K) inhibitor, and PD 098059 (mitogen-activated/extracellular signal-regulated kinase (MEK) inhibitor). Cells which were administered GLP-2 exhibited more than 10-fold increase in proliferation, a response which was blunted by Genistein, LY294002, and PD 098059 in a dose-dependent fashion. Such a response was attributed at least in part to both the PI 3-kinase and the MAPK pathways [104].

Further studies in humans are warranted in order to elucidate the underlying mechanisms pertinent in gut proliferation mediated by both GLP-1 and GLP-2. However, the experimental models provide clear evidence of the involvement of the enteroendocrine system in intestinal proliferation.

## 6. The “Microbial Hypothesis” in Inflammation of Adipocyte Dysfunction 

### 6.1. Limitations of the Classical Hypothesis of Adipocyte Dysfunction

In light of the previously discussed viral infection and bacterial endotoxaemia, the conventional perspective of adipocyte dysfunction in the pathogenesis of obesity clearly exhibits a number of limitations in substantially explaining adipocyte inflammation as a feature of obesity pathogenesis. Firstly, the trigger for inflammatory cell recruitment and secretion of inflammatory cytokines in adipose tissue is poorly accounted for, with no experimental evidence in existence to the best of our knowledge [105–107]. Secondly, inflammation in obesity is described as being “sterile” in nature [105]. No consideration has therefore been given to the contributory role of microbes or microbial endotoxins such as bacterial LPS in initiating and perpetuating chronic low grade inflammation.

### 6.2. Postulated Inflammatory Triggers of Adipocyte Dysfunction

The physiological goal of inflammation is primarily to eliminate an insult which the body deems alien and noxious. This is often employed via the innate and subsequently the adaptive immune responses should the insult persist. The acute inflammatory response is therefore converted to chronic inflammation in the setting of a persistent insult [107]. It is therefore not unreasonable to propose that viral infection and metabolic endotoxaemia may serve as triggers of inflammation that underpin adipocyte dysfunction. Mechanistically, we have subdivided possible triggers of inflammation into “transient” and “persistent” factors on the basis of chronicity.

#### 6.2.1. Transient Inflammatory Triggers

In the case of viral infection, it is plausible to suggest that acute repetitive infections may serve as a transient factor that regularly initiates acute inflammation. This is later converted to chronic inflammation by background persistent factors. The infective situation may be described as a repetitive acute-on-chronic scenario. Repetitive infections may be explained by the fact that obesity is associated with an increased susceptibility to infection due to impaired systemic immune function [108].

#### 6.2.2. Persistent Inflammatory Triggers

The chronic nature of dysbiosis and subsequent metabolic endotoxaemia is in keeping with the chronicity of inflammation observed in obesity and may qualify as persistent factors. This may therefore suggest a critical role for dysbiosis and endotoxaemia in initiating and perpetuating chronic inflammation via the persistent presence of bacterial LPS in the systemic circulation. The repetitive acute-on-chronic viral infections along with chronic dysbiosis and metabolic endotoxaemia may therefore possibly act in a concerted manner to promote chronic inflammation.

### 6.3. Determinants of Low Grade Inflammatory Tone

The characteristically low grade “subclinical” tone of inflammation in obesity can also be accounted for. The resident gut microflora is an essential component of gastrointestinal physiology and its myriad role is beyond the scope of this review. A degree of immunological tolerance is exerted so as not to instigate an immune response against them, in order to preserve their critical function [109, 110]. It has been suggested that the gut microbiome regulates not only local intestinal immunity but also systemic immunity [111]. It is therefore logical to suggest that the impaired systemic immune function that occurs in obesity may at least in part be attributed to dysbiosis and metabolic endotoxaemia, therefore resulting in subclinical inflammation.

### 6.4. The Adipocyte as an Immune Cell

It has been demonstrated that immature haematopoietic stem cells are present in adipocytes, and mature adipocytes express membrane-bound NADPH oxidase similar to that of phagocytic neutrophils [112]. It has also been suggested that the fact that adipocytes and their progenitors share a common embryonic origin with cells of the immune system rationalises the adipogenic response to endotoxaemia (i.e., adipocyte expansion) [108]. It is therefore plausible to propose that adipocyte hypertrophy and hyperplasia (i.e., the obesity response) are akin to an immune response mounted against an exogenous antigen, in terms of immune cell replication and phagocytosis. Therefore, perhaps the effects of the often recommended healthy diet and exercise extend far beyond lipolysis and weight loss. The beneficial effects of such measures may in addition restore a healthy microbiome and thus eliminate dysbiosis and endotoxaemia as triggers of obesity. Exercise-induced hyperaemia and angiogenesis may serve to increase circulatory transport of repair factors to regions of gut barrier damage, promoting repair and resolution.

The “microbial hypothesis” attempts to address the limitations of the classical hypothesis in explaining the trigger of inflammation in adipocyte dysfunction, and the possible role that microbes and microbial endotoxins may play in the process. Rather than refute the classical hypothesis altogether, this hypothesis acts to supplement the classical hypothesis. It does so in terms of filling in the missing link in the chain of events, taking into account the aforementioned recent experimental data that have undoubtedly suggested a critical role for microbes and microbial products in obesity pathobiology.

## 7. Conclusion

Taken together, there is undoubtedly an association between viral infection and metabolic endotoxaemia in the pathogenesis of obesity. However, proof of association, no matter how appealing or robust, does not necessarily imply causation. Since the obesity epidemic is recognised as being multifactorial in origin, a multifaceted approach to management is therefore warranted. Infection treatment and prevention do not formally feature in clinical practice guidelines given the fact that basic science research in “infectobesity” is still at its infancy, yet very promising. However, one should make no mistake to undermine the overwhelming evidence in both animal models and humans that suggest an important role for infection and endotoxaemia in the development of obesity. In fact, this is gaining prominence so much so that Massachusetts General Hospital is sponsoring the Faecal Microbiota Transplant for Obesity and Metabolism clinical trial, the first clinical trial to study the impact of gut bacteria on weight (https://clinicaltrials.gov/ct2/show/NCT02530385). The trial is currently at recruitment stage and due to commence soon. The results of this trial are awaited anxiously by the scientific community, as it could possibly cause a paradigm shift in our understanding of obesity and alter the management course of this epidemic.

## Figures and Tables

**Table 1:** Meta-analyses on the relationship between human adv-36 infection and obesity development in humans.

Study	Number of cases	Population size	Study end points	Major findings
Xu et al. [17]	(i) 9 cross-sectional studies, 9 case-control studies, and 6 cohort studies	(i) 10191 study subjects including adults and children	(i) HAdV-36 infection rate in obese and lean groups (ii) BMI level and BMI *z*-score in HAdV-36 positive and negative groups	(i) HAdV-36 infection increased the risk of obesity (ii) HAdV-36 also increased the risk of weight gain in adults, which was not observed in children

Shang et al. [18]	(i) 11 case control studies	(i) 5739 study subjects including adults and children	(i) HAdV-36 infection and obesity risk	(i) HAdV-36 infection is associated with an increased risk of obesity development (ii) Risk is increased in children and those with a BMI of ≥30 kg/cm^2^

Yamada et al. [19]	(i) 10 cross-sectional studies	(i) 2870 study subjects including adults and children	(i) Evaluating the association between HAdV-36 infection and obesity/metabolic markers	(i) HAdV-36 infection is associated with the risk of obesity and weight gain, but not with abnormal metabolic markers including waist circumference
